# Validation of CRownLab Pneumoplex for the Detection of *Klebsiella pneumoniae* and *Acinetobacter baumannii* Using RT‐PCR in Patients With Sepsis at a Tertiary Hospital in Indonesia

**DOI:** 10.1155/ijm/8576494

**Published:** 2026-02-09

**Authors:** Fadrian Fadrian, Linosefa Linosefa, Keesa Nabila Afida, Disa Hijratul Muharramah, Vyora Ulvyana, Paishal Mizan, Vidola Yasena Putri

**Affiliations:** ^1^ Department of Internal Medicine, Division of Tropical Medicine and Infectious Disease, Dr. M. Djamil General Hospital, Padang, Indonesia; ^2^ Department of Internal Medicine, Division of Tropical Medicine and Infectious Disease, Faculty of Medicine, Universitas Andalas, Padang, Indonesia, unand.ac.id; ^3^ Department of Microbiology, Faculty of Medicine, Universitas Andalas, Padang, Indonesia, unand.ac.id; ^4^ Department of Research and Development, Dr. M. Djamil General Hospital, Padang, Indonesia; ^5^ Department of Epidemiology, Faculty of Public Health, Universitas Sriwijaya, Palembang, Indonesia, unsri.ac.id; ^6^ Department of Internal Medicine, Faculty of Medicine, Universitas Andalas, Padang, Indonesia, unand.ac.id; ^7^ Faculty of Medicine, Universitas Andalas, Padang, Indonesia, unand.ac.id

**Keywords:** bacterial infections, molecular diagnostic techniques, real-time polymerase chain reaction, sensitivity and specificity, sepsis

## Abstract

**Objectives:**

Early diagnosis of bacterial sepsis remains a significant challenge in clinical practice, primarily due to the limitations of conventional methods such as blood culture, which require prolonged turnaround times and have low sensitivity. The real‐time polymerase chain reaction (RT‐PCR) method using CRownLab Pneumoplex was developed to enable rapid and simultaneous detection of pathogens. This study is aimed at evaluating the diagnostic validity of CRownLab Pneumoplex in detecting *Klebsiella pneumoniae* and *Acinetobacter baumannii* in patients with sepsis at a tertiary hospital in Indonesia.

**Methods:**

This cross‐sectional study involved 400 sepsis patients recruited between October 2024 and February 2025. Blood, sputum, urine, and pus specimens were collected from patients who met the inclusion and exclusion criteria. All samples were analyzed using culture methods as the gold standard and RT‐PCR using CRownLab Pneumoplex. Sensitivity, specificity, positive predictive value (PPV), negative predictive value (NPV), and diagnostic accuracy were calculated.

**Results:**

RT‐PCR detection of *K. pneumoniae* demonstrated a sensitivity of 89.0%, specificity of 87.7%, PPV of 65.2%, NPV of 96.9%, and accuracy of 88.0%. For *A. baumannii*, sensitivity was 92.9%, specificity was 88.9%, PPV was 23.2%, NPV was 99.7%, and accuracy was 89.0%. Overall, combined detection yielded a sensitivity of 88.9%, specificity of 87.0%, PPV of 69.3%, NPV of 96.0%, and diagnostic accuracy of 87.5%. For sputum specimens, sensitivity was 73.5%, specificity was 96.9%, PPV was 98.9%, NPV was 50.0%, and accuracy was 78.5%. The mortality rate among sepsis patients in this study was 69.5%.

**Conclusions:**

The RT‐PCR method using CRownLab Pneumoplex demonstrated high diagnostic validity for the detection of *K. pneumoniae* but only moderate reliability for *A. baumannii*. For sputum specimens, the RT‐PCR method showed good diagnostic performance in patients with sepsis and respiratory infections.

## 1. Introduction

Sepsis is a global health threat associated with high mortality [1]. It ranks among the leading global causes of morbidity and mortality, ranking within the top 10 causes of death overall and as the second most common cause in noncardiac intensive care units (ICUs). A tertiary hospital study reported that 40.7% of ICU patients experienced severe sepsis, whereas 59.3% developed septic shock [2]. Mortality among ICU sepsis patients remains significant, ranging from 25% to 40% [3].

A 2021 cohort study identified *Escherichia coli* as the most frequent sepsis pathogen (21.5%), followed by *Klebsiella pneumoniae* (9.0%), methicillin‐sensitive *Staphylococcus aureus* (6.5%), and *Streptococcus pneumoniae* (5.0%) [4]. Meanwhile, antibiogram data from Dr. M. Djamil General Hospital in 2024 showed that the most frequently isolated pathogens in inpatients were *K. pneumoniae* (1,376 isolates), *E. coli* (583), *Pseudomonas aeruginosa* (424), *Acinetobacter baumannii* (315), and *Staphylococcus haemolyticus* (298) [5].

The 2023 Update on Sepsis and Septic Shock in Adults highlights the importance of prompt and appropriate antibiotic administration following sepsis recognition as critical to improving outcomes [6]. Iyengar et al. showed that each hour of delay in antimicrobial therapy increases mortality risk by 7.6%, emphasizing the urgency of treatment in septic shock [7].

These diagnostic limitations have encouraged the use of biomarkers such as leukocyte count, C‐reactive protein (CRP), and procalcitonin, although their diagnostic performance remains inconsistent due to limited sensitivity and specificity [8]. Molecular diagnostic methods such as real‐time polymerase chain reaction (RT‐PCR) have been developed to detect bacterial deoxyribonucleic acid **(**DNA) directly from blood samples, providing results in approximately 3–4 hours [9]. RT‐PCR enables real‐time quantification of pathogen DNA during the amplification process. DNA amplification is visualized by the increase in fluorescence intensity from fluorogenic probes, which is proportional to the amount of amplified DNA [10].

Several studies have demonstrated the effectiveness of RT‐PCR in detecting pathogens responsible for sepsis. Zhao et al. [11] found that digital PCR (dPCR) detected pathogens in 73.9% of cases, compared to 27.5% with blood cultures. The sensitivity of dPCR was 63.2%, suggesting it offers superior diagnostic performance in sepsis. To improve diagnostic efficiency and reduce turnaround time, multiplex PCR (mPCR) was developed to detect multiple pathogens and antimicrobial resistance genes in a single test. This allows for earlier and more targeted antimicrobial therapy, particularly important in critical care settings where prompt intervention improves survival and shortens hospital stays. PCR has limitations as a diagnostic test, particularly its inability to distinguish between live and dead cells, which can result in false‐positive results in individuals who are not infected with the pathogen [12].

CRownLab Pneumoplex is one of the multiplex RT‐PCR kits designed to identify bacterial pathogens in respiratory infections. CRownLab Pneumoplex is a domestically developed and assembled RT‐PCR kit with a working principle equivalent to previously validated multiplex RT‐PCR kits, enabling greater cost efficiency. This study is aimed at evaluate the diagnostic accuracy of CRownLab Pneumoplex for early and reliable detection of bacterial sepsis and its potential contribution to improved patient outcomes, as well as its low‐cost advantages as a sustainable diagnostic solution in resource‐limited settings.

## 2. Materials and Methods

### 2.1. Study Design

This cross‐sectional study was conducted at Dr. M. Djamil General Hospital, Padang, on October 2024. Sepsis patients from various inpatient wards, including internal medicine and infectious disease units, were enrolled through consecutive sampling. A total of 400 samples were collected between October 2024 and February 2025. In this study, we collected biological fluids, including blood, sputum, urine, and pus specimens.

### 2.2. Study Population and Sampling

The study focused on patients diagnosed with sepsis who were treated at Dr. M. Djamil General Hospital beginning in October 2024. Participants were selected based on predefined inclusion and exclusion criteria. Eligibility required a Sequential Organ Failure Assessment (SOFA) score of ≥ 2, a confirmed diagnosis of an infectious disease, and an adequate specimen volume for both culture and PCR testing, with informed consent obtained. All bacterial species included in this study are clinically relevant pathogens associated with sepsis. Exclusion criteria included nonbacterial causes of sepsis, such as viral or fungal infections, or discharge against medical advice.

### 2.3. Specimen Collection and Diagnostic Testing

Specimens for microbiological analysis were collected using standardized sterile procedures. Urine samples were obtained by aspirating through a Foley catheter using a sterile syringe under aseptic conditions. Sputum samples were collected using a sterile mucus extractor connected to suction under aseptic conditions. The collected specimens originated from the lower respiratory tract and exhibited mucous or mucopurulent characteristics. Samples that met the criteria for good‐quality sputum defined by the presence of thick, opaque mucus with minimal salivary contamination were included for further molecular analysis. Wound or surgical site infections were sampled by swabbing the lesion directly, ensuring the swab did not come into contact with the periwound skin, and then placing the sample into sterile transport tubes. Blood cultures were acquired by drawing venous blood from two anatomically distinct sites using sterile 10‐mL syringes and depositing the samples into BacT/ALERT 3D culture bottles (BioMérieux). All collected specimens were promptly delivered to the Microbiology Laboratory of Dr. M. Djamil General Hospital for processing and identification using the VITEK 2 Compact automated system.

In certain clinical situations, specimen collection was adapted. Blood cultures alone were taken when sampling from the infection site was not feasible. In contrast, site‐specific cultures were prioritized if blood samples had already been collected during the onset of fever and before the administration of antibiotics. For molecular analysis, DNA extraction was performed using the CRownLab Bacterial Sputum Extraction Kit, followed by detection with the CRownLab Pneumoplex RT‐PCR assay. RT‐PCR utilizes specific hybridization between a probe and a target sequence, followed by the activity of DNA polymerase, which produces a fluorescent signal. The RT‐PCR reaction was prepared by assembling a premix consisting of 4 *μ*L of CRownLab Pneumoplex 3.0 RT‐PCR Mixture and 12 *μ*L of Pneumoplex 3.0 PP Mixture A/B, yielding a total premix volume of 16 *μ*L per reaction. This mixture was dispensed into each reaction tube, followed by the addition of 4 *μ*L of a DNA sample, a positive control, or a negative control, resulting in a final reaction volume of 20 *μ*L. RT‐PCR amplification was performed using the following thermal protocol: an initial predenaturation at 95°C for 3 min, followed by 40 cycles of denaturation at 95°C for 5 s, followed by annealing and extension at 53°C for 30 s. In this kit, detection of *K. pneumoniae* targets the gapA gene, while detection of *A. baumannii* targets the ompA gene.

### 2.4. Data Collection and Variables

Demographic and clinical data were recorded, including comorbidities, source of infection, and culture results. Laboratory tests were analyzed in the microbiology and molecular laboratories. The primary comparison was between the diagnostic performance of molecular PCR and conventional culture, using culture as the gold standard.

### 2.5. Statistical Analysis

All data were analyzed using SPSS version 25.0 (IBM Corp., Armonk, New York, United States). Descriptive statistics were used to summarize baseline characteristics. Diagnostic test accuracy was evaluated by calculating sensitivity, specificity, PPV, NPV, and overall accuracy using 2 × 2 contingency tables, with conventional culture serving as the reference standard.

### 2.6. Ethical Considerations

The study protocol was approved by the Institutional Review Board of Dr. M. Djamil General Hospital, Padang. All participants or their legal representatives provided written informed consent prior to inclusion in the study. Confidentiality of patient data was maintained in accordance with ethical research standards (Approval Number: DP.04.03/D.XVI.XI/247/2024).

## 3. Results

This study included 400 sepsis patients who met the inclusion and exclusion criteria and were hospitalized at Dr. M. Djamil General Hospital, Padang, between October 2024 and February 2025. Baseline characteristics are presented in Table 1. Most participants were male (57.25%) and aged over 60 years (54%). Community‐acquired pneumonia (CAP) was the most frequent source of infection (61%), followed by hospital‐acquired pneumonia (HAP) (33.75%) and skin and soft tissue infections (SSTIs) (13%). Septic shock occurred in 41.5% of cases, with a mean SOFA score of 5.70 ± 2.97 (range: 2–17).

**Table 1:** Baseline characteristics of the study population.

Characteristics	Value
Gender, *n* (%)	
Male	229 (57.25)
Female	171 (42.75)
Age, *n* (%)	
18–60 years	184 (46)
> 60 years	216 (54)
Infectious disease diagnosis, *n* (%)	
Community‐acquired pneumonia (CAP)	244 (61)
Hospital‐acquired pneumonia (HAP)	135 (33.75)
Urinary tract infection (UTI)	13 (3.25)
Skin and soft tissue infections (SSTI)	52 (13)
Intraabdominal infection (IAI)	12 (3)
Septic shock, *n* (%)	
Yes	166 (41.5)
No	234 (58.5)
Mean SOFA score (± standard deviation)	5.70 (2.97)
Comorbidities, *n* (%)	
Diabetes mellitus	106 (26.5)
Chronic kidney disease	84 (21)
Chronic lung disease	32 (8)
Chronic liver disease	27 (6.75)
Heart failure	74 (18.5)
Malignancy	86 (21.5)
Human immunodeficiency virus (HIV)	12 (3)
Neutropenia	4 (1)
Initial empirical antibiotic therapy, *n* (%)	
Ampicillin–sulbactam	181 (45.25)
Cefepime	148 (37)
Ceftazidime	9 (2.25)
Meropenem	26 (6.5)
Levofloxacin	6 (1.5)
Moxifloxacin	2 (0.5)
Cefoperazone–sulbactam	16 (4)
Cefotaxime	10 (2.5)
Bacteria identified from culture	
Blood culture, (*n* = 234) *n* (%)	
*Klebsiella pneumoniae*	8 (3.41)
*Acinetobacter baumannii*	0 (0)
*Staphylococcus aureus*	2 (0.85)
*Burkholderia cepacia*	1 (0.42)
*Escherichia coli*	3 (1.28)
*Enterobacter cloacae*	1 (0.42)
*Enterococcus faecalis*	1 (0.42)
*Pseudomonas aeruginosa*	1 (0.42)
*Photobacterium damselae*	1 (0.42)
*Sphingomonas paucimobilis*	2 (0.85)
*Staphylococcus caprae*	1 (0.42)
*Staphylococcus cohnii spp urealyticus*	2 (0.85)
*Staphylococcus epidermidis*	3 (1.28)
*Staphylococcus haemolyticus*	13 (5.55)
*Staphylococcus hominis*	5 (2.13)
*Staphylococcus kloosii*	1 (0.42)
*Staphylococcus warneri*	2 (0.85)
*Staphylococcus xylosus*	3 (1.28)
No growth	181 (77.35)
Sputum Culture, (*n* = 150) *n* (%)	
*Klebsiella pneumoniae*	74 (49.33)
*Acinetobacter baumannii*	14 (9.33)
*Staphylococcus aureus*	1 (0.66)
*Escherichia coli*	12 (8)
*Enterobacter aerogenes*	1 (0.66)
*Enterobacter cloacae*	2 (1.33)
*Enterococcus faecalis*	1 (0.66)
*Pseudomonas aeruginosa*	3 (2)
*Pseudomonas luteola*	1 (0.66)
*Staphylococcus haemolyticus*	4 (2.66)
No growth	37 (24.66)
Urine Culture, (*n* = 11) *n* (%)	
No growth	11 (100)
Pus Culture, (*n* = 5) *n* (%)	
No growth	5 (100)
Bacteria Identified by polymerase chain reaction (PCR)	
Blood Specimen, (*n* = 234) *n* (%)	
*Klebsiella pneumoniae*	4 (1.70)
*Acinetobacter baumannii*	0 (0)
Sputum Specimen, (*n* = 150) *n* (%)	
*Klebsiella pneumoniae*	105 (70)
*Acinetobacter baumannii*	53 (35.33)
Urine Specimen, (*n* = 11) *n* (%)	
*Klebsiella pneumoniae*	2 (18.18)
*Acinetobacter baumannii*	0 (0)
Pus Specimen, (*n* = 5) *n* (%)	
*Klebsiella pneumoniae*	2 (40)
*Acinetobacter baumannii*	1 (20)
History of hospitalization in the past 60 days, *n* (%)	
Yes	149 (37.25)
No	251 (62.75)
Referral from another healthcare facility, *n* (%)	
Yes	190 (47.5)
No	210 (52.5)
Prior antibiotic use, *n* (%)	
Yes	199 (49.75)
No	201 (50.25)
Discharge outcome, *n* (%)	
Survived	122 (30.5)
Died	278 (69.5)
Length of hospital stay, *n* (%)	
<7 days	113 (28.25)
7–14 days	142 (35.5)
15–28 days	104 (26)
> 28 days	41 (10.25)

Comorbidities were common, with diabetes mellitus being the most prevalent (26.5%), followed by malignancy (21.5%) and chronic kidney disease (21%). The presence of multiple comorbidities reflects a high disease burden and increased vulnerability to severe infections. Empirical antibiotic choices were dominated by ampicillin–sulbactam (45.25%) and cefepime (37%). Among 234 blood culture samples, *K. pneumoniae* was the most frequently identified organism (3.41%). Sputum cultures (*n* = 150) also showed predominance of *K. pneumoniae* (49.66%), followed by *A. baumannii* (9.39%). PCR detection yielded higher pathogen identification rates, particularly in sputum specimens: *K. pneumoniae* (69.79%) and *A. baumannii* (35.57%). In blood samples, PCR detected *K. pneumoniae* in only 1.7% of cases. PCR also identified pathogens in urine and pus samples, whereas culture methods failed to detect organisms in these specimens.

Previous antibiotic use was reported in 49.75% of patients, and 37.25% had been hospitalized within the past 60 days. Additionally, 47.5% were referred from other healthcare facilities. The overall mortality rate was 69.5%, whereas 30.5% of patients were discharged alive. Length of stay varied, with most patients hospitalized for 7–14 days (35.5%), followed by < 7 days (28.25%), 15–28 days (26%), and > 28 days (10.25%).

This study evaluated the diagnostic performance of RT‐PCR using the CRownLab Pneumoplex kit for the detection of two major sepsis‐causing pathogens: *K. pneumoniae* and *A. baumannii*, using microbial culture as the reference standard. Additionally, a pooled analysis was conducted to assess the overall accuracy of the RT‐PCR method in clinical settings. As shown in Table 2, RT‐PCR demonstrated a sensitivity of 89.0% and specificity of 87.7%, with a PPV of 65.2% and a high NPV of 96.9% for *K. pneumoniae*. The overall accuracy was 88.0%. These results indicate that RT‐PCR reliably identified *K. pneumoniae* cases (high sensitivity) and effectively excluded true‐negative cases (high NPV). However, the moderate PPV suggests a relatively high proportion of false positives, warranting further confirmation via culture.

**Table 2:** Comparison of diagnostic performance of RT‐PCR with CRownLab Pneumoplex and culture methods for key pathogens associated with sepsis.

Bacteria	Sensitivity (%)	Specificity (%)	PPV (%)	NPV (%)	Accuracy (%)
*Klebsiella pneumoniae*	89.0	87.7	65.2	96.9	88.0
*Acinetobacter baumannii*	92.9	88.9	23.2	99.7	89.0

For *A. baumannii*, RT‐PCR exhibited strong diagnostic performance, with a sensitivity of 92.9% and specificity of 88.9%. Although the PPV was relatively low at 23.2%, the NPV reached 99.7%, indicating that negative results were highly reliable. The accuracy of 89.0% supports the validity of RT‐PCR as a screening tool. The low PPV may be attributed to the low prevalence of *A. baumannii* in the study population, which statistically reduces the proportion of true positives among PCR‐positive results.

As summarized in Table 3, the pooled analysis of all targeted pathogens revealed an overall sensitivity of 88.9% and specificity of 87.0%. The PPV was 69.3%, indicating that the majority of positive results represented true infections, whereas the high NPV of 96.0% further supports the method′s strength in excluding bacterial infection. The overall diagnostic accuracy of 87.5% highlights the potential of RT‐PCR using the CRownLab Pneumoplex kit as a valuable adjunct for early pathogen detection in sepsis management.

**Table 3:** Comparison of diagnostic performance of RT‐PCR with CRownLab Pneumoplex compared with culture for combined pathogen variants.

Pathogen	Sensitivity (%)	Specificity (%)	PPV (%)	NPV (%)	Accuracy (%)
All bacteria	88.9	87.0	69.3	96.0	87.5

In Table 4, the diagnostic values of RT‐PCR using CRownLab Pneumoplex on sputum specimens showed a sensitivity of 73.5%, specificity of 96.9%, PPV of 98.9%, NPV of 50.0%, and accuracy of 78.52%. Sputum specimens represented the majority of infection focus diagnoses in this study. RT‐PCR demonstrated reliable potential as a diagnostic tool, particularly for sputum specimens. The PPV value reaching 98.8% indicates that positive results are true positives.

**Table 4:** Comparison of the diagnostic performance of RT‐PCR using CRownLab Pneumoplex and culture methods for sputum specimens.

Specimens	Sensitivity (%)	Specificity (%)	PPV (%)	NPV (%)	Accuracy (%)
Sputum	73.5	96.9	98.9	50.0	78.52

## 4. Discussion

The majority of study participants were male (57.25%) and aged ≥ 60 years (54%). This aligns with findings by Fortini et al. [13] who reported a higher incidence of sepsis among men in Italy. In contrast, Fataya et al. [14] found a higher risk among women in Indonesia, suggesting that gender‐related sepsis prevalence may vary by population and environmental context. Biologically, hormonal and immune differences may contribute to these observed disparities. Estrogen, for example, modulates CD4+ and CD8+ T cell responses through cytokine regulation, potentially enhancing both protection and inflammatory risk [15].

In this study, most sepsis patients were aged over 60. Aging impairs immune function, reducing leukocyte response and altering cytokine production. The 2020 WHO Global Report on Sepsis described a biphasic incidence, peaking in neonates and the elderly, highlighting age‐related vulnerability [16]. This is largely due to immunosenescence, marked by reduced T and B cell function, impaired phagocyte activity, and a shift from naïve to memory T cells, diminishing the ability to respond to new infections [17–20].

CAP was the most frequent cause of sepsis in this study (61%), followed by HAP (33.75%) and SSTIs (13%). At Dr. M. Djamil General Hospital, Decroli et al. [21] found that 78.4% of sepsis cases were linked to respiratory infections. In this study, 41.5% of sepsis patients developed septic shock, with an average SOFA score of 5.70 ± 2.97, indicating significant early organ dysfunction. A cohort study of 250 ICU patients reported a median SOFA score of 7 at admission, with 29.4% progressing to septic shock within 24 h, reflecting rapid clinical deterioration [22]. Optimal sepsis management includes infection control, hemodynamic stabilization, and modulation of the host response. Failure to promptly address these components can result in progression to septic shock and death [23].

Comorbidities among sepsis patients in this study were varied, with many presenting two or more conditions, reflecting a high disease burden and increased vulnerability to severe infections. Diabetes mellitus was the most frequent comorbidity (26.5%), followed by malignancy (21.5%) and chronic kidney disease (21%). This aligns with a retrospective cohort study at Dr. M. Djamil General Hospital, which found Type 2 diabetes in 36.5% of sepsis patients [24]. Elevated blood glucose levels impair neutrophil function, including chemotaxis, phagocytosis, microbial killing, and increase the production of proinflammatory cytokines such as *tumor necrosis factor-alpha* (TNF‐*α*), *interleukin-6* (IL‐6), and *interleukin-1β* (IL‐1*β*). These changes contribute to a weakened defense against infection and worsen the inflammatory response, leading to poorer clinical outcomes in sepsis [14].

The most frequently used initial empirical antibiotics were ampicillin–sulbactam (45.25%) and cefepime (37%), both broad‐spectrum agents targeting gram‐negative and gram‐positive bacteria. This is consistent with findings by Fadrian et al. [25] who reported cefepime–levofloxacin (44.6%) as the most common empirical combination, followed by ceftriaxone–levofloxacin (8.4%). These prescribing patterns align with guidelines from the Infectious Diseases Society of America and the American Thoracic Society, particularly for managing CAP and HAP, which were the predominant sources of sepsis in this study. The 2019 guidelines recommend a beta‐lactam plus macrolide, or monotherapy with respiratory fluoroquinolones such as levofloxacin or moxifloxacin for CAP. For HAP, the 2016 guidelines advise broad‐spectrum antibiotics, including cefepime, meropenem, or ceftazidime, particularly for patients at risk of multidrug‐resistant organisms or with high mortality risk [26, 27].

In this study, blood cultures from 234 sepsis patients revealed *K. pneumoniae* as the most common isolate (3.41%). Similarly, sputum cultures from 150 samples showed a predominance of *K. pneumoniae* (49.33%), followed by *A. baumannii* (9.33%). The low positivity rate of blood cultures is consistent with findings by Yana K et al. [28] who reported a 24% positivity rate in sepsis patients at Hasan Sadikin Hospital, likely due to challenges in isolating microorganisms from blood samples. Fadrian et al. [24] also identified *K. pneumoniae* (55.6%) and *E. coli* (44.4%) as the most frequent blood pathogens in sepsis, with sputum cultures similarly dominated by *K. pneumoniae* (78.3%). However, other studies report different trends. Sieswerda et al. [29] found *E. coli* (23%) and *S. aureus* (10%) as the leading blood isolates, whereas Rhee et al. [30] also reported *E. coli* as the most common, followed by *S. aureus* and *Streptococcus* spp., with sputum cultures dominated by *S. aureus*, *Pseudomonas aeruginosa*, and *Streptococcus* spp.

The predominance of *K. pneumoniae* in culture results may be attributed to the high proportion of lower respiratory tract infections, particularly CAP, as the primary source of sepsis. *K. pneumoniae* is a key CAP pathogen, especially in patients with risk factors such as advanced age and comorbidities. The high prevalence of diabetes mellitus in this study may also contribute to increased susceptibility to gram‐negative infections, including *K. pneumoniae*. Chronic hyperglycemia impairs immune cell phagocytic function, enhances bacterial adhesion to respiratory epithelium, and exacerbates immune dysfunction, promoting colonization and infection by opportunistic pathogens [31, 32].

Pathogen detection using PCR in sepsis patients showed higher positivity rates than conventional culture, particularly in sputum specimens. The most frequently identified organisms were *K. pneumoniae* (70%) and *A. baumannii* (35.33%). In blood samples, *K. pneumoniae* was detected in only 1.70%. PCR also identified bacterial DNA in urine and pus samples, despite limited specimen numbers, whereas conventional cultures yielded no growth in these samples.

A study by Chang et al. comparing blood culture and multiplex RT‐PCR for detecting sepsis pathogens demonstrated that mPCR had a broader detection range. It identified 18 positive cases, whereas blood culture detected only seven, all of which were also identified by PCR. These results indicate that mPCR is a promising method for early diagnosis of acute sepsis, offering faster results within approximately 2 h, which is significantly quicker than traditional blood cultures [33].

The higher pathogen detection sensitivity of PCR, particularly in patients with CAP as the primary source of infection, can be attributed to several factors. First, PCR detects microbial DNA or RNA directly from clinical samples without requiring culture growth, enabling pathogen identification even after prior empirical antibiotic therapy. Second, CAP is often caused by pathogens that are difficult to culture or have slow growth, such as *K. pneumoniae* or *Streptococcus pneumoniae*, making PCR more effective for rapid and accurate detection. Third, sputum from lower respiratory tract infections contains a high microbial load, which enhances the sensitivity of molecular techniques [34–36].

In this study, 49.75% of sepsis patients had a history of prior antibiotic use, and 37.25% had been hospitalized within the previous 60 days. This aligns with findings by Fadrian et al. [37] who reported prior antibiotic use in 73.5% of hospitalized sepsis patients. Previous antibiotic exposure and hospitalization are key risk factors for sepsis due to their role in promoting colonization by resistant bacteria and disrupting the normal microbiota. Such conditions favor the emergence of resistant pathogens like *K. pneumoniae*, *A. baumannii*, and *Pseudomonas aeruginosa*, commonly associated with nosocomial infections and increased sepsis‐related mortality [38]. In this study, 47.5% of sepsis patients were referred from other healthcare facilities. The high referral rate reflects limited diagnostic and therapeutic capacity, as well as a lack of trained personnel, in lower‐level facilities to manage severe sepsis. As a medical emergency, sepsis requires rapid, comprehensive treatment, including hemodynamic monitoring, intravenous antibiotics, and intensive care access. Therefore, early‐diagnosed or suspected cases are commonly referred to tertiary hospitals with more advanced resources and specialized care [39, 40].

Sepsis is a life‐threatening organ dysfunction caused by severe infection and remains a leading cause of death globally. Without timely intervention, mortality rates can reach 30 to 35% [41]. In this study, the mortality rate among sepsis patients was notably high at 69.5%, with only 30.5% surviving to discharge. Although sepsis is often considered the direct cause of death, some evidence suggests that outcomes may be more closely related to underlying comorbidities, which may not be preventable solely by treating sepsis [42].

In this study, most sepsis patients were hospitalized for 7–14 days (35.5%), followed by less than 7 days (28.25%), 15–28 days (26%), and more than 28 days (10.25%). This aligns with findings by Kim et al. who reported a median length of stay of 15.9 days in patients diagnosed with sepsis before admission, compared with 5.5 days in those diagnosed during hospitalization [43]. Length of hospital stay in sepsis patients is influenced by disease severity, delayed diagnosis, response to initial therapy, complications such as septic shock or multiorgan failure, and comorbidities like diabetes and chronic kidney disease. Many patients require intensive care, vasopressors, and mechanical ventilation, which extend hospitalization [44, 45].

Research on the use of mPCR for detecting bacterial sepsis remains limited. However, several studies have demonstrated its high sensitivity and specificity in identifying pathogens across various infections, including respiratory, diarrheal, and gastrointestinal diseases. Yun Li et al. [46] evaluated mPCR combined with membrane biochip assay for detecting nine sepsis‐related pathogens, including *A. baumannii*, *E. coli*, *K. pneumoniae*, *Pseudomonas aeruginosa*, *Enterococcus faecalis*, *S. aureus*, *Staphylococcus epidermidis*, *Streptococcus pneumoniae*, and *Candida albicans*. Among 179 clinical samples, the biochip detected pathogens in 36 samples (20.11%) compared with 33 samples (18.44%) by blood culture.

The use of mPCR in managing severe pneumonia in ICUs has improved the accuracy of empirical antibiotic therapy and clinical outcomes. A multicenter observational study in Morocco reported that mPCR achieved 96.9% sensitivity and 92% specificity. Its implementation increased antibiotic appropriateness from 38.7% to 67% and was associated with reduced ICU mortality. However, despite faster pathogen identification, some studies found no significant impact on 30‐day mortality or ICU length of stay. A retrospective study in Colombia reported that mPCR use in severe pneumonia cases did not significantly alter antibiotic duration or short‐term mortality [47, 48].

In this study, RT‐PCR CrownLab Pneumoplex demonstrated good diagnostic performance in detecting *K. pneumoniae*, with a sensitivity of 89.0% and specificity of 87.7%. The high NPV of 96.9% indicates reliable exclusion of negative cases. However, the lower PPV of 65.2% suggests a higher likelihood of false positives, potentially due to disease prevalence, cross‐contamination, or detection of nonviable DNA. These findings are consistent with Poritz et al. [49] who reported a sensitivity of 96.7% and NPV of 99.6%, with a lower PPV of 17.4% for *K. pneumoniae* using mPCR.

In detecting *A. baumannii*, RT‐PCR demonstrated high performance, with a sensitivity of 92.9% and specificity of 88.9%. The NPV was excellent at 99.7%, confirming the reliability of negative results. However, PPV was low at 23.2%, likely due to the low prevalence of *A. baumannii* in the study population, as PPV is highly influenced by disease prevalence. Luyt et al. [50] reported that the Unyvero mPCR system had a sensitivity of 81% and specificity of 99% for ventilator‐associated pneumonia pathogens, including *A. baumannii*, with faster detection than conventional culture. Overall, RT‐PCR including CRownLab Pneumoplex serves as a rapid and sensitive screening tool for detecting sepsis pathogens, particularly in lower respiratory tract infections such as CAP. However, positive results should be interpreted with caution and confirmed through culture or clinical assessment, especially in populations with low prevalence of the pathogen.

In sputum specimens, the diagnostic performance of RT‐PCR demonstrated a sensitivity of 73.5%, specificity of 96.8%, PPV of 98.9%, NPV of 50.0%, and accuracy of 78.52%. Sputum specimens represented the most frequent source of infection in sepsis patients in this study, specifically CAP. Early detection in sepsis patients with a primary infection focus from the respiratory tract is beneficial for guiding therapeutic decisions in sepsis management [51]. Source control of the infectious focus has been associated with improved survival in recent observational and randomized cluster studies. Source control must be achieved as soon as possible following initial resuscitation, as delays beyond 6–12 h have been linked to significant decreases in survival [52]. Additionally, multiplex RT‐PCR demonstrated comparable capability in detecting pathogens as singleplex RT‐PCR, which served as the accredited diagnostic test [53].

This study has several limitations. The use of a cross‐sectional design restricts the ability to assess changes over time, treatment outcomes, or the prognostic value of RT‐PCR in the clinical course of sepsis. Furthermore, the diagnostic validation of the CRownLab Pneumoplex kit was conducted exclusively at a single tertiary care center, Dr. M. Djamil General Hospital, Padang, which may not represent pathogen profiles or healthcare capacities in other regions or institutions. This study presents a novel diagnostic tool that enables rapid detection of sepsis pathogens and is more cost‐effective than the mPCR platforms currently implemented in other tertiary hospitals. Another limitation of this study is that multiple specimens were collected from each patient without determining which specimen type yielded the most reliable diagnostic result.

## 5. Conclusion

RT‐PCR using CRownLab Pneumoplex showed good diagnostic performance for *K. pneumoniae*, with sensitivity of 89.0%, specificity of 87.7%, and accuracy of 88.0%. Its high NPV (96.9%) supports a high ability to rule out infection. Detection of *A. baumannii* also showed high sensitivity (92.9%) and NPV (99.7%), but low PPV (23.2%) suggests possible false positives due to low prevalence. Overall, RT‐PCR showed good validity for early screening of bacterial sepsis. The method is especially useful for detecting *K. pneumoniae* but only moderate reliability for *A. baumannii*. Its shorter turnaround time (3–4 h) compared with culture (48–72 h) enables faster clinical decisions and more appropriate empirical antibiotic use. Broad implementation may improve sepsis management, reduce delays in therapy, and support antimicrobial stewardship. From a cost perspective, the CRownLab RT‐PCR assay is more affordable than standard multiplex RT‐PCR platforms used in other tertiary hospitals, particularly in resource‐limited or developing countries.

## Funding

This work was supported by Dr. M. Djamil General Hospital.

## Ethics Statement

This study was approved by the Health Research Ethics Committee of the Dr. M. Djamil Central General Hospital, Padang, Indonesia, in accordance with national and institutional ethical standards for human research (Approval Number: DP.04.03/D.XVI.XI/247/2024).

## Conflicts of Interest

The authors declare no conflicts of interest.

## Data Availability

The data supporting the findings of this study are available from the corresponding author upon reasonable request. Due to privacy and ethical considerations, the data are not publicly accessible.
